# Chemometric Approach for Discriminating the Volatile Profile of Cooked Glutinous and Normal-Amylose Rice Cultivars from Representative Japanese Production Areas Using GC × GC-TOFMS

**DOI:** 10.3390/foods14152751

**Published:** 2025-08-06

**Authors:** Takayoshi Tanaka, Junhan Zhang, Shuntaro Isoya, Tatsuro Maeda, Kazuya Hasegawa, Tetsuya Araki

**Affiliations:** 1 Graduate School of Agriculture and Life Sciences, University of Tokyo, Tokyo 113-003, Japan; t-takayoshi@g.ecc.u-tokyo.ac.jp (T.T.); zhang-junhan@g.ecc.u-tokyo.ac.jp (J.Z.); aaraki@g.ecc.u-tokyo.ac.jp (T.A.); 2 Department of Health and Dietetics, Teikyo Heisei University, Tokyo 170-8445, Japan; s.isoya0705@gmail.com (S.I.); k.hasegawa@thu.ac.jp (K.H.)

**Keywords:** GC × GC-TOFMS, japonica rice, glutinous (waxy) rice, volatile organic compounds, functional group profiling, chemometric analysis

## Abstract

Cooked-rice aroma strongly affects consumer choice, yet the chemical traits distinguishing glutinous rice from normal-amylose japonica rice remain underexplored because earlier studies targeted only a few dozen volatiles using one-dimensional gas chromatography–mass spectrometry (GC-MS). In this study, four glutinous and seven normal Japanese cultivars were cooked under identical conditions, their headspace volatiles trapped with MonoTrap and qualitatively profiled by comprehensive GC × GC-TOFMS. The two-dimensional platform resolved 1924 peaks—about ten-fold previous coverage—and, together with hierarchical clustering, PCA, heatmap visualization and volcano plots, cleanly separated the starch classes (78.3% cumulative PCA variance; Euclidean distance > 140). Volcano plots highlighted 277 compounds enriched in the glutinous cultivars and 295 in Koshihikari, including 270 compounds that were not previously documented in rice. Normal cultivars were dominated by ethers, aldehydes, amines and other nitrogenous volatiles associated with grainy, grassy and toasty notes. Glutinous cultivars showed abundant ketones, furans, carboxylic acids, thiols, steroids, nitro compounds, pyrroles and diverse hydrocarbons and aromatics, yielding sweeter, fruitier and floral accents. These results expand the volatile library for japonica rice, provide molecular markers for flavor-oriented breeding and demonstrate the power of GC × GC-TOFMS coupled with chemometrics for grain aroma research.

## 1. Introduction

Rice (*Oryza sativa* L.) is a global dietary staple, providing over half the world’s population with approximately 21% of their caloric intake [1]. In East Asia, consumers prefer japonica rice because of its moderate elasticity and stickiness, which are linked to its low amylose content [2]. Within this subspecies, cultivars bifurcate into normal and glutinous (waxy) types, the latter being revered for its sweet, floral aroma and use in traditional dishes [3,4].

The aroma of rice is a critical driver of consumer acceptance; however, the molecular underpinnings of rice aroma remain only partially elucidated. Glutinous cultivars typically release higher levels of unsaturated aldehydes and medium-chain ketones such as 2-heptanone, which impart creamy and fruity nuances originating from lipid oxidation and Maillard-derived pathways [5]. In contrast, normal-amylose japonica tends to accumulate C6 aldehydes such as hexanal, which imparts fresh-cut grass notes, together with nitrogen-containing volatiles derived from amino acid catabolism that evoke earthy or cereal-like impressions [6,7]. These compositional contrasts suggest fundamentally different biochemical fluxes between the two rice types. However, most comparative investigations rely on conventional one-dimensional gas chromatography (1D-GC). 1D-GC offers limited peak capacity; therefore, it cannot fully resolve the hundreds of partially co-eluting compounds found in cooked rice headspace, leading to quantitative bias and the loss of low-abundance yet odor-active constituents [8]. Consequently, a more powerful separation technology is required to capture the complete volatile fingerprints that distinguish glutinous from normal rice.

Comprehensive two-dimensional GC (GC × GC) coupled with time-of-flight MS (TOFMS) offers transformative potential. By employing orthogonal columns and thermal modulation, GC × GC-TOFMS achieves a peak capacity that is 5–10 times higher than that of 1D-GC, resolves co-eluting compounds and detects trace analytes [9]. This approach has revolutionized metabolomics in complex plant and biofluid matrices [10,11,12] and the environmental monitoring of trace contaminants [13], but its power remains untapped for rice aroma research. Despite the documented aroma differences between glutinous and normal rice, no study has used GC × GC-TOF-MS to simultaneously profile and compare the complete volatile spectra of both types of rice. Moreover, the functional group distributions that govern the reactivity, volatility and sensory impact remain unclassified. Clarifying these distributions is essential for elucidating the biochemical pathways underlying the rice aroma. Thus, the volatile metabolites that distinguish glutinous rice from normal rice are still poorly characterized at the functional group level, and the full analytical power of GC × GC-TOFMS is yet to be exploited in rice flavor research.

The objective of this study was to qualitatively profile the functional group landscape of volatile compounds that Japanese consumers actually encounter when eating cooked glutinous and non-glutinous japonica rice. To capture these real-world differences, representative cultivars grown in their characteristic production areas were analyzed by untargeted GC × GC-TOFMS and interpreted with multivariate techniques across contrasting amylose classes. Eleven widely cultivated Japanese varieties were examined: four glutinous (Kitayukimochi, Tatsukomochi, Hiyokumochi and Kazenoko-mochi) and seven normal-amylose varieties (Akitakomachi, Masshigura, Nanatsuboshi, Hitome-bore, Sasanishiki, Hinohikari and Koshihikari). Leveraging the high separation efficiency of GC × GC and the rapid, high-resolution detection of TOFMS, this study provides a comprehensive volatile catalogue, assigns compounds to functional classes and identifies markers that discriminate glutinous rice from normal rice by comparing normalized peak area patterns within this Japanese cultivar set.

This work is exploratory and qualitative, aiming to reveal broad compositional trends without constructing predictive calibration models. The resulting insights deepen our understanding of rice flavor chemistry and support breeding and quality-assessment efforts targeting consumer-preferred aroma attributes.

## 2. Materials and Methods

### 2.1. Sample Preparation

Eleven Oryza sativa L. (japonica type) cultivars harvested in 2021–2022, of which four were glutinous and seven were normal-amylose varieties drawn from Japan’s major rice-growing regions, were analyzed, with each cultivar prepared in triplicate (Table 1). All samples were kept in the dark at 22 ± 2 °C until processing. For each batch, 800 g of polished rice was weighed and washed with running tap water. Washing began with 3 min of gentle stirring, continued with two rapid rinses to remove surface starch and finished with three 1 min rounds of circular rubbing to ensure thorough cleaning. After the water was drained, fresh water at 18 °C was added at 1.35 times the rice weight, and the grains were soaked for 60 min.

Cooking was performed in an IH rice cooker (Panasonic SR-STS18VC-W (Panasonic Operation, Osaka, Japan), 6-stage IH, maximum output 1400 W, inner-pot volume ≈ 3 L). The quick room program (25 min) consists in an initial 220 steam-injection phase (3 s), a boiling plateau at 100 and a 5 min hold, followed by a 10 min resting stage. The rice cooker heats the entire pot wall and phases through the IH coils. Upon completion, the rice was fluffed with chopsticks using the Sharikiri method and then held in the cooker (warm, mode, 70 °C) for 10 min to equalize the moisture. The total weight was recorded to determine the cooking yield. Cooked rice (20 g) was sampled from the central layer, avoiding the top 0.5 cm to minimize evaporative losses, and then equilibrated at room temperature (22 ± 1 °C) for 3 h prior to analysis. All procedures were standardized and performed using calibrated instruments: a thermometer, digital balance, graduated beaker, timer and fine-mesh strainer.

### 2.2. Volatile Extraction Using MonoTrap

Headspace vials (40 mL, precision-thread; GERSTEL GmbH & Co. KG, Mülheim an der Ruhr, Germany) were sealed with silver aluminum caps fitted with PTFE/silicone septa to ensure an airtight environment. Volatiles were trapped using a MonoTrap RGPS-TD sorbent (Cat. No. 1050-74202, GL Sciences, Tokyo, Japan; 2.9 mm Ø × 10 mm). This hybrid cartridge consists in a silica-monolith framework that is chemically modified and embedded with graphite carbon and end-capped polydimethylsiloxane (PDMS). It is interconnected through a mesoporous network (>150 m^2^ g^−1^ surface area) that provides high adsorption capacity and efficient thermal desorption, making it well suited for capturing a wide range of headspace volatiles.

For VOC sampling, 20 g of cooked rice was transferred to a 40 mL Clean Pinhole Septum vial (GERSTEL). The sealed vial was equilibrated at 25 °C for 60 min in a temperature-controlled room (23 ± 2 °C). The two MonoTraps were then suspended in the headspace and exposed for 180 min to collect the volatiles released from the rice, aiming to minimize spatial bias within the vial and increase adsorption capacity, thereby providing a more stable and sensitive capture of rice volatiles.

### 2.3. GC × GC-TOFMS Analysis

Volatile compounds were analyzed using a LECO Pegasus BT4D GC × GC–TOFMS (LECO Corporation, Saint Joseph, MI, USA) coupled to an Agilent 8890A gas chromatograph (Agilent Technologies, Palo Alto, CA, USA) and operated using a GERSTEL MPS RoboticPro autosampler (GERSTEL GmbH & Co. KG, Mülheim an der Ruhr, Germany). The thermal desorption unit (TDU2) was held at 30 °C for 0.5 min, ramped at 720 °C min^−1^ to 300 °C and held for 5 min. Desorbed analytes were passed into a cooled-injection system (CIS4) maintained at −50 °C for 1.5 min and then heated at 12 °C s^−1^ to 240 °C and held for 20 min; the TDU–CIS transfer line was kept at 300 °C throughout.

Chromatographic separation was performed using a polar primary column (InertCap Pure-WAX, 30 m × 0.25 mm i.d., 0.25 µm film, GL Sciences, Tokyo, Japan) [14] and a non-polar secondary column (InertCap 5MS/NP, 1.3 m × 0.18 mm i.d., 0.18 µm film, GL Sciences, Tokyo, Japan) [15] linked by a dual-stage cryogenic modulator housed in an auxiliary oven, following GL Sciences technical guidance for food aroma analysis [7,8]. The primary oven program started at 40 °C (1 min), rose to 100 °C at 10 °C min^−1^ and then to 250 °C at 5 °C min^−1^ and was held 10 min. The secondary oven and modulator were offset by +5 °C and +20 °C, ending at 255 °C and 270 °C, respectively. A 5.5 s modulation period (two 1.65 s hot-pulse jets separated by a 1.10 s cold-pulse interval) was chosen empirically to avoid wrap-around yet fully resolve steamed-rice volatiles. Helium (99.999 % purity) served as the carrier gas at a constant 1.0 mL min^−1^. The electron ionization TOF-MS was operated with a 250 °C ion source, 255 °C transfer line and 70 eV ionization energy and acquired spectra from *m*/*z* 35–600 at 200 Hz (detector voltage 2049 V). Relative abundances were obtained from total ion chromatograms (TIC) following standard metabolite-profiling practices. A blank extract was used to identify and eliminate system-derived artifacts. Peaks likely associated with column bleeding or instrument-related contaminants were reduced by excluding compounds containing terms such as “glycol”, “silan”, “siloxan” or “crown” from the dataset. In addition, compound names were manually reviewed to remove obvious duplicates or inconsistent entries. These steps were intended to minimize the inclusion of instrumental noise and improve the likelihood that the remaining 3421 chromatographic peaks represented meaningful VOC features.

### 2.4. Chromatographic and Statistical Analysis

Data were acquired and processed with LECO ChromaTOF v 5.54.48.070156 (LECO Corporation, St. Joseph, MI, USA). Qualitative identification combined external retention-index calibration and spectral-library matching: a C_9_–C_40_ n-alkane mixture standard in n-hexane (cat. no. 1021-58325, GL Sciences, Tokyo, Japan; 50 µg mL^−1^ each) was analyzed under identical GC × GC-TOF-MS conditions to establish reference 1D and 2D retention times. To maximize discovery of low-abundance and previously unreported VOCs—and thus create a comprehensive baseline dataset for future omics-based or targeted studies—mass spectra were matched to the NIST 20 and Wiley 11 libraries using a deliberately lower similarity threshold (≥550). All resulting identifications are therefore tentative. Peak areas were normalized within each sample by dividing each individual peak area by the sample’s total peak area, yielding peak area percentages. These values were used solely to visualize relative abundances and are reported as mean ± SD for three biological replicates; no absolute quantification was performed.

Exploratory analyses, including principal component analysis (PCA), hierarchical clustering (HCA) and heatmap visualization, were performed using JMP Pro 17 (JMP Statistical Discovery, Cary, NC, USA). HCA uses Euclidean distances and Ward linkages to group samples by volatile compound profiles, whereas heatmaps depict inter-VOC correlations. Statistical significance between groups was assessed using Student’s *t*-test and one-way ANOVA; *p* < 0.05 was considered as a preliminary indicator of potential difference. Differentially expressed VOCs were further screened in R 4.4.0 by volcano plot analysis, by applying the fold-change (FC) and *t*-test criteria. A feature was deemed potentially differential when log_2_(FC) ≥ 1 or ≤–1 in combination with *p* < 0.05. These plots and thresholds were used exclusively for exploratory prioritization and do not constitute definitive quantitative claims.

## 3. Results and Discussion

**Figure 1:**
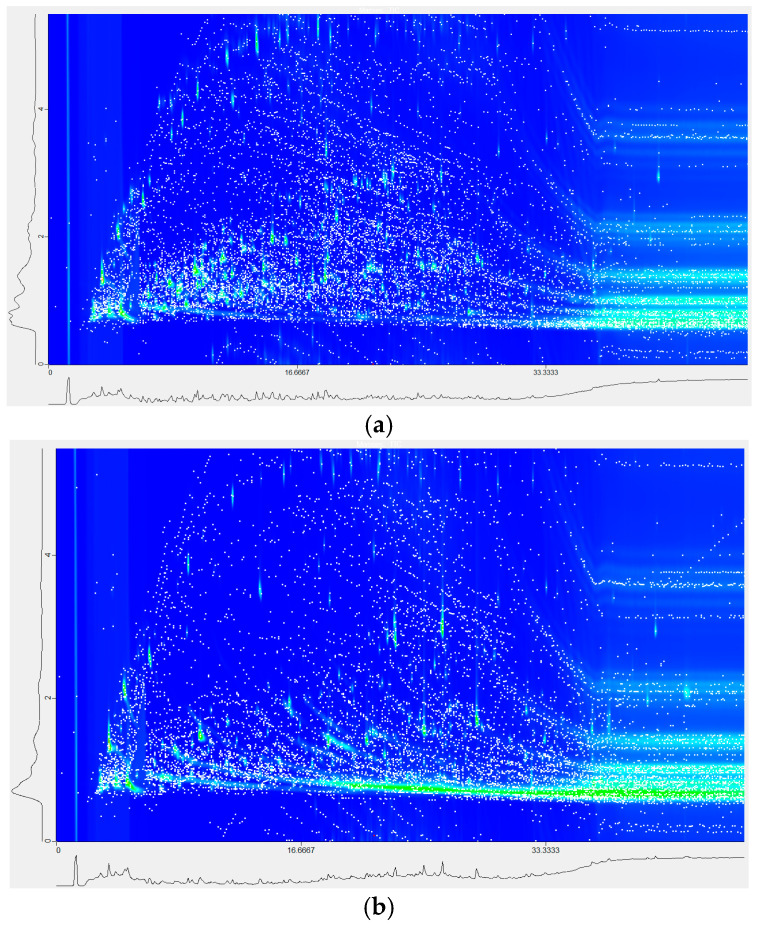
Representative GC × GC chromatogram of cooked-rice volatiles. (**a**) *Koshihikari* (*normal-amylose*); (**b**) *Kazenokomochi* (*glutinous*). The abscissa shows first-dimension retention time (1D), and the ordinate shows second-dimension retention time (2D). Each dot corresponds to a detected VOC feature. Across all samples, 3421 peaks were detected; 1924 (56.2%) were tentatively matched to NIST 20/Wiley 11 libraries at a similarity cut-off of 550, leaving 1497 peaks (43.8%) unidentified.

Comprehensive GC × GC–TOF-MS revealed 3421 peaks, of which 1924 were tentatively identified by matching to the NIST 20 and Wiley 11 spectral libraries (see representative chromatogram in Figure 1a,b). The resulting compounds include alkanes, alkenes, alkynes, aldehydes, ketones, alcohols, ethers, amines, carboxylic acids, arenes and steroids and oxygen-, nitrogen- and sulfur-containing heterocycles. These findings align with previous characterizations of cooked rice volatiles [16,17,18] while revealing several functional group classes not previously reported, underscoring the higher resolving power of GC × GC–TOFMS.

Among the seven normal amylose cultivars analyzed, Koshihikari served as a reference for direct comparison with the four glutinous varieties because of its extensive cultivation, consistent quality traits and well-characterized volatile profiles. The remaining six normal cultivars were retained to provide the diversity required for multivariate analyses (hierarchical clustering, PCA and heatmap visualization) that captured inter-varietal variance. Consequently, Section 3.1, Section 3.2 and Section 3.3 use all normal cultivars to define group-level patterns, whereas Section 3.4 and Section 3.5 concentrate on Koshihikari versus the glutinous group, aligning with this study’s aim of delineating varietal differences in volatile and functional group profiles.

### 3.1. HCA Analysis

**Figure 2:**
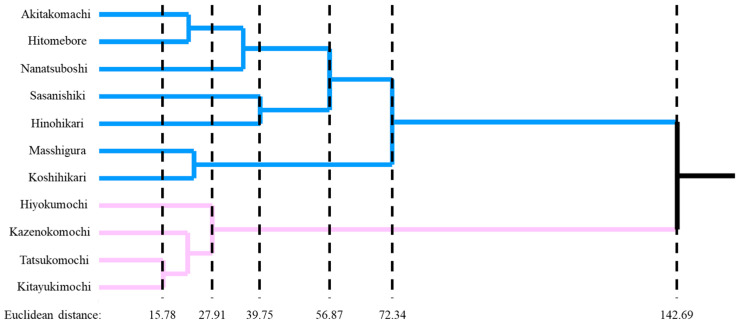
Exploratory hierarchical cluster analysis (HCA) of of 11 japonica rice cultivars based on volatile profiles. Blue lines indicate normal-amylose cultivars, and pink lines indicate glutinous cultivars. Dashed vertical lines represent the Euclidean distance scale used for clustering.

The hierarchical cluster analysis dendrogram (Figure 2) separated the samples into two main branches: one containing glutinous cultivars and the other containing normal amylose cultivars. These branches diverged at a Euclidean distance greater than 140, indicating a pronounced difference in volatile profiles and, by extension, in grain quality between glutinous and non-glutinous rice. The glutinous rice cultivars displayed only small distances from one another, with a maximum Euclidean distance of 27.9, indicating a high degree of similarity in their volatile components. By contrast, the normal-amylose cluster showed greater internal dispersion, with a maximum Euclidean distance of 72.3, reflecting broader variability in their volatile composition. This pattern suggests that glutinous varieties possess more conserved and uniform aroma profiles than normal amylose rice varieties, likely because they share genetic traits linked to starch type and common metabolic pathways that generate volatiles.

### 3.2. PCA Analysis

**Figure 3:**
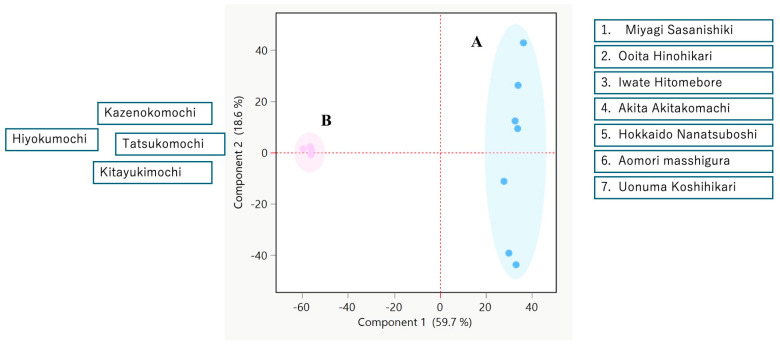
PCA score plot showing the variance in the glutinous rice and non-glutinous rice. Rice samples are clustered into the following two groups: non-glutinous rice samples are marked in blue (A); glutinous rice samples are marked in pink (B).

Figure 3 depicts the principal component analysis (PCA) of the volatile profiles of 11 rice cultivars, each measured in biological triplicate; the corresponding relative standard deviation (RSD) values are provided in Appendix A, Table A1. The first two principal components explain 78.3% of the total variance (PC1, 59.7%; PC2, 18.6%), yielding a clear two-dimensional separation of the samples. The glutinous cultivars cluster tightly, underscoring the high similarity of their volatile compositions and showing no statistically significant differences (*p* > 0.05).

Normal amylose cultivars were more widely dispersed, indicating greater heterogeneity in both composition and abundance of volatiles. In particular, the normal cultivar 7 plotted at a considerable distance from the glutinous cultivar 4, suggesting a distinctive volatile signature. These patterns mirror the groupings revealed by hierarchical clustering and reinforce the conclusion that glutinous rice possesses a more conserved aroma profile, whereas normal rice exhibits broader compositional diversity.

### 3.3. Heatmap Analysis

**Figure 4:**
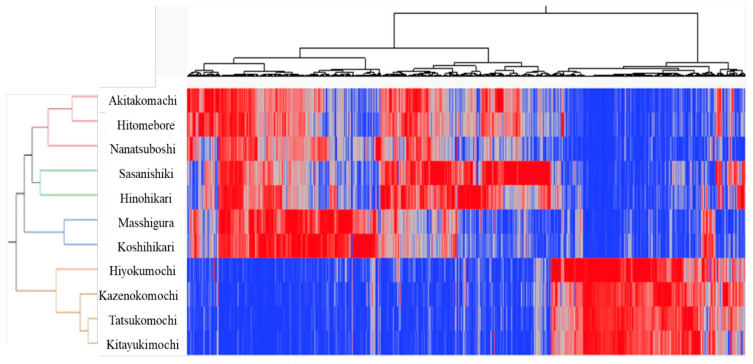
Exploratory heatmap of relative VOC compound-class abundances.

Heatmap analysis of the 1924 tentatively identified VOCs (Figure 4) divides the cultivars into four clear clusters: one containing Akitakomachi, Hitomebore and Nanatsuboshi; a second with Sasanishiki and Hinohikari; a third grouping Masshigura and Koshihikari; and a fourth consisting of the glutinous varieties Hiyokumochi, Kazenokomochi, Tatsukomochi and Kitayukimochi. The first three clusters represent all normal-amylose cultivars (about 70% of the dataset) and display broadly similar, though internally stratified, volatile profiles, whereas the glutinous cluster (about 30%) is tightly grouped. A pronounced red block in the lower-right quadrant of the map, comprising 796 VOC features, marks compounds that are abundant in glutinous rice but largely depleted in the normal sets and provides the main chemical contrast corroborated by PCA and HCA.

### 3.4. Volcano Plot Analysis

Zeng et al. [19] identified a suite of volatiles regarded as markers of glutinous rice including hexanal, 2-pentylfuran, (E)-2-heptenal, 1-hexanol, nonanal, 1-octen-3-ol, (E)-2-nonenal, (E,Z)-2,4-decadienal, (E,E)-2,4-decadienal, γ-nonalactone, 2-pentadecanone, 2-methoxy-4-vinylphenol, 4-vinylphenol, indole and vanillin. In the present study, only hexanal was detected. Compounds such as 1-octen-3-ol, γ-nonalactone, indole, vanillin and other vinylphenol derivatives were not detected, a discrepancy that may reflect cultivar differences or methodological factors. 1-octen-3-ol and indole volatilize within the first minutes of steaming and drop below detection when headspace is collected after cooling [20], and cultivar genetics also matter; indole, for instance, appeared only in Nipponbare and not in five other types of glutinous rice analyzed under identical conditions [21].

Among the volatiles detected in this study, 2-pentylfuran, (E)-2-heptenal, benzaldehyde, (E)-2-nonenal, (E,E)-2,4-nonadienal and (E,E)-2,4-decadienal showed comparable abundance in both rice groups. In contrast, geranyl acetone, previously linked to glutinous rice, displayed lower levels in the glutinous cultivars analyzed, suggesting a possible influence of cultivar background or starch-type metabolism on its formation.

**Figure 5:**
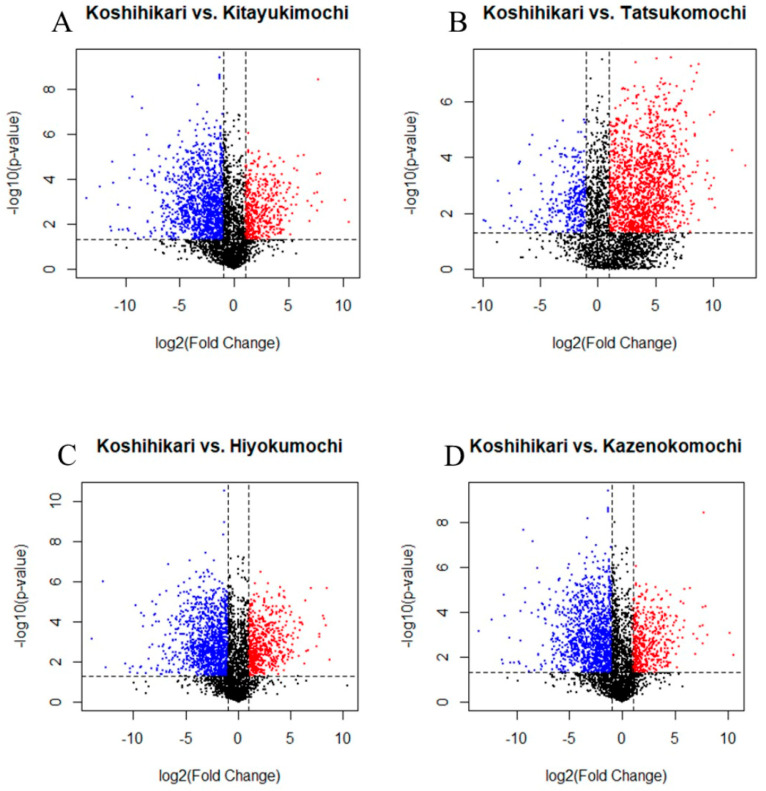
The volcano plot analysis of Koshihikari against four types of glutinous rice with the *x*-axis showing log_2_ fold change and the *y*-axis displaying −log_10_ *p* values from *t*-tests. Red dots indicate compounds significantly upregulated in glutinous rice and blue dots represent compounds enriched in Koshihikari. (**A**) Koshihikari vs. Kitayukimochi; (**B**) Koshihikari vs. Tatsukomochi; (**C**) Koshihikari vs.Hiyokumochi; (**D**) Koshihikari vs. Kazenokomochi.

Figure 5 presents a volcano plot comparing the volatile profiles of the four glutinous cultivars with that of Koshihikari, representing the normal-amylose group. The plots highlight significant differences in volatile compounds, with the *x*-axis showing log_2_ fold change and the *y*-axis displaying −log_10_ *p* values from *t*-tests. Red dots indicate compounds that are significantly upregulated in glutinous cultivars, particularly Kitayukimochi, and are associated with fruity, floral and creamy aromas (e.g., ketones and esters). In contrast, the blue dots represent compounds enriched in Koshihikari, linked to grassy, toasty and earthy notes (e.g., aldehydes and alcohols). Non-significant compounds (gray/black dots) showed no marked differences between groups. The full lists of the top 50 significantly enriched compounds for each group are provided in Appendix A Table A2 and Table A3. These findings align with those of previous studies, reinforcing that glutinous rice favors sweet and floral volatiles, whereas normal rice produces earthier aromas, highlighting the influence of starch type on aroma profiles.

Among the glutinous cultivars, distinct metabolic patterns emerged compared with Koshihikari. Kitayukimochi exhibited 272 volatiles enriched in Koshihikari, whereas 617 volatiles were more abundant in Koshihikari. Tatsukomochi showed the greatest disparity, with 916 elevated volatile compounds compared to 206 in Koshihikari. Similarly, Hiyokumochi and Kazenokomochi displayed 332 and 246 upregulated volatiles, respectively, compared with 580 and 583 in Koshihikari. Collectively, these results highlight the unique volatile signatures of each glutinous cultivar, reflecting metabolic divergence from normal-amylose rice. Further analysis revealed 279 glutinous-specific volatiles (157 identified), whereas Koshihikari produced 295 cultivar-specific compounds (119 identified). Notably, no overlap occurred between the glutinous- and Koshihikari-specific volatiles, emphasizing a clear compositional divide between the two rice types.

A comparison with prior studies provides this context. Zeng et al. [19] listed 96 glutinous-specific volatiles; seven, 1-dodecanol, 1-heptanol, 1-hexadecanol, 2-nonanone, hexanal, octanal and octanoic acid, were detected. In contrast, none of the 14 glutinous markers proposed by Ajarayasiri et al. [22] appeared in this dataset. Among the 22 compounds associated with glutinous rice reported by Fukuda et al. [23], hexanal and octanal were observed. Hu et al. [24] highlighted 20 glutinous-enriched volatiles, nine of which were deemed aroma-impactful. Only hexanal was confirmed in our samples. The findings for normal rice aligned with those for cooked non-fragrant japonica reported by Zhao et al. [25] and Zhao et al. [26], who identified heptanal, 2-heptanone and 1-octen-3-ol as discriminative lipid oxidation products that are also abundant in Koshihikari. The occurrence of 1-octen-3-ol may be influenced by the steaming protocol, which could promote the release or formation of lipid-derived volatiles [27]. Beyond the scope of the present study, future studies should clarify their impact through controlled experiments separating thermal effects from cultivar differences.

Odor-active-value analysis by Yang et al. [28] showed that hexanal, octanal and heptanal drove the sensory separation of glutinous rice from normal rice, giving glutinous cultivars a greener and mushroom-like note, whereas normal rice contained the same aldehydes at a far lower intensity. Likewise, Chen et al. [29] reported higher levels of hexanal, octanal, nonanal and (E)-2-heptenal in glutinous rice, attributing the hexanal abundance to a low amylose content that limits V-complex formation, as well as a comparatively high concentration of 1-pentanol in normal rice, reflecting its simpler, lower-intensity VOC profile.

We detected 270 volatile compounds that have not been previously reported in glutinous rice, 148 of which were tentatively identified through matches with the NIST 20 and Wiley 11 libraries. In contrast, the volatile characteristics of glutinous rice were virtually absent in Koshihikari, in which only 1-nonanol was detected. These results highlight the unique volatile repertoire of glutinous cultivars and distinguish their aroma profiles from those of normal amylose rice.

### 3.5. Functional Groups Analysis

**Figure 6:**
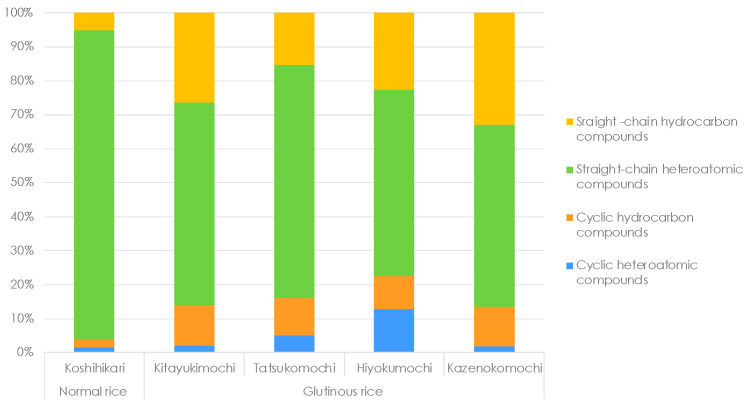
Relative distribution of four broad chemical classes of cultivar-specific volatile organic compounds in cooked normal rice (Koshihikari) and glutinous rice (Kitayukimochi, Tatsukomochi, Kazenokomochi and Hiyokumochi).

Compounds were grouped into four broad classes (Figure 6): linear hydrocarbons, cyclic hydrocarbons, linear heteroatomic compounds containing O, N or S and cyclic heteroatomic compounds with the same heteroatoms. A finer breakdown of the functional groups is shown in Figure 6.

Figure 6 indicates that Koshihikari contains a lower proportion of cyclic hydrocarbons and a higher proportion of linear heteroatomic compounds than the glutinous cultivars. The glutinous group showed no overall pronounced differences among the cultivars, although Hiyokumochi displayed a modest increase in cyclic hydrocarbon content compared with the other glutinous varieties.

**Figure 7:**
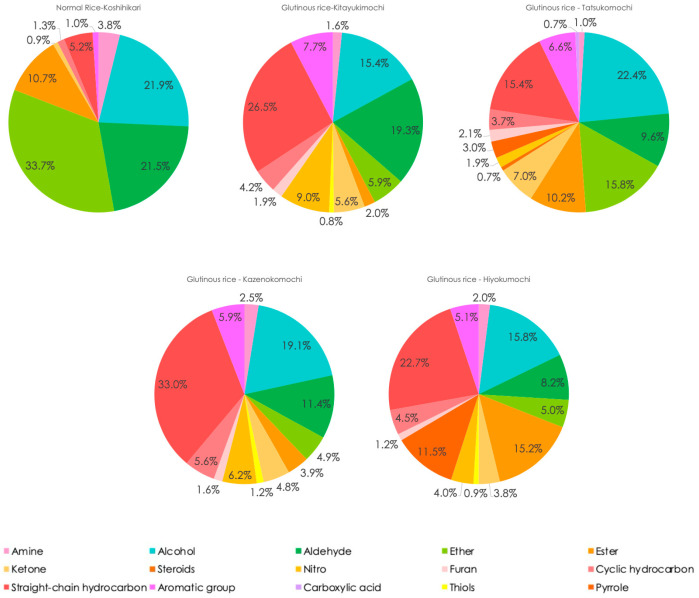
Functional group composition of cultivar-specific volatile organic compounds in cooked normal rice (Koshihikari) and glutinous rice (Kitayukimochi, Tatsukomochi, Kazenokomochi and Hiyokumochi) in % pie chart.

Our functional group survey shows a clear partitioning between the two rice types. Normal-amylose cultivars contain considerably more ethers, aldehydes and amines, whereas glutinous cultivars display a broader functional group palette—including steroids, nitro compounds, thiols, furans, carboxylic acids and pyrroles—and markedly higher levels of ketones, straight and cyclic hydrocarbons and aromatic compounds (Figure 7).

Although we detected substantially higher ether concentrations in normal-amylose rice, earlier reports rarely cite ethers as discriminating or aroma-active compounds. This discrepancy is best explained by methodological contrast. Previous works have depended on one-dimensional GC with static-headspace or solvent extraction, privileging very volatile aldehydes and alcohols. Under such conditions, semi-volatile ethers tend to co-elute, give poor spectra and are relegated to unknown peaks. Our study instead couples MonoTrap headspace sorptive extraction with GC × GC-TOFMS where the MonoTrap selectively enriches medium-chain ethers, and the orthogonal, cryo-modulated separation increases peak capacity five- to ten-fold, allowing these compounds to be fully resolved and confidently annotated. The greater ether signal in our data therefore reflects superior extraction and chromatographic resolution rather than a genuine absence of ethers in glutinous rice.

Chen et al. [29] reported higher aldehyde concentrations in glutinous rice, whereas our study found that normal rice, particularly Koshihikari, was richer in aldehydes such as hexanal, heptanal and nonanal, as well as alcohols such as 1-pentanol and 1-hexanol, which contribute to grassy and toasty sensory characteristics. This difference may stem from variations in data presentation. Chen et al. [29] reported absolute VOC concentrations, whereas our analysis was based on relative percentages. Given that glutinous rice generally has a higher total VOC content, this could explain the contrasting results. We also detected amines in both rice types, with normal rice displaying greater abundance, which is an observation not fully reported previously.

Our data also separate the two rice classes by steroids, nitro compounds and thiols—differences that have received little attention in earlier work. Hu et al. [20] likewise reported a larger overall VOC pool in glutinous rice, including aldehyde concentrations roughly double those of normal rice. They highlighted 2-pentylfuran as especially abundant and aroma-active in glutinous samples, and we observed the same trend, with furans absent from normal rice, suggesting this compound contributes the buttery nuance characteristic of glutinous cultivars. Carboxylic acids followed a similar pattern, as we detected them only in waxy rice, and Hu et al. [24] identified octanoic and butanoic acids as major fatty and cheesy contributors in the same group.

Elevated levels of pyrrole-type volatiles in glutinous rice—especially indole—have also been reported. Our data agree that the glutinous cultivars Tatsukomochi and Hiyokumochi contained detectable pyrroles, whereas none were found in normal rice. Indole, a member of the pyrrole functional group, is recognized as a potent sweet-floral odorant and thus a key contributor to the characteristic aroma of glutinous rice [22].

Ketones appear in both rice classes, yet few studies have compared their levels directly. These volatiles arise chiefly from the thermal degradation of fatty acids. In our dataset, glutinous rice yielded higher ketone percentages, likely because its nearly amylose-free starch gelatinizes quickly, retains more moisture and thereby accelerates lipid hydrolysis and β-oxidative cleavage during steaming. Commonly reported ketones, including 6-Methyl-5-hepten-2-one, 2-Heptanone and acetone, may contribute to the sweet and fruity sensory attributes in glutinous rice [30].

Our results are consistent with those of Yang et al. [28], who observed that glutinous rice contains higher levels of straight-chain hydrocarbons, such as heptane, octane and nonane, which are likely derived from lipid oxidation. This difference may stem from the elevated total fat and unsaturated fatty acid contents of certain glutinous cultivars and low amylose content facilitated lipid hydrolysis. Aromatic compounds showed the same bias where benzaldehyde and 2-methoxy-4-vinylphenol were more abundant in glutinous rice, most likely generated via amino acid degradation and ferulic acid decarboxylation during the Maillard reaction, imparting almond-like and sweet-vanilla notes. In contrast, normal rice exhibits a greater abundance of nitrogen-containing volatiles and ethers, which contribute to its characteristic toasty, grassy and pungent aroma. These findings reinforce the critical role of the functional group composition in determining rice aroma and quality.

Although glutinous cultivars share many volatile compounds, their functional group distributions vary significantly, highlighting their compositional diversity even within the same starch type. A similar trend exists among normal japonica rice varieties. Zeng et al. [31] found that esters and indole were prominent in Koshihikari and Akitakomachi but nearly absent in Nihonbare. Such variation suggests that functional group profiles are both cultivar-specific and influenced by starch type (glutinous vs. non-glutinous) as well as the enzymatic reactions that occur during cooking. Overall, the volatile class patterns observed in our study were basically consistent with those of prior research with several new functional groups identified, reinforcing the reliability of our compound identification and functional group assignments.

Notably, Zhou et al. [32] stressed that concentration alone does not determine sensory relevance. Aldehydes such as hexanal and nonanal may be abundant, yet they contribute only modestly to perceived aroma. Because GC × GC-TOFMS yielded ~2000 peaks per sample, we reported only concentrations, since generating odor-activity values (OAVs) for each peak would have required extensive confirmation and sensory testing beyond this study’s scope. In addition, all compound identifications were qualitative, based on spectral matching (≥550) without external standards, and approximately 43.8% of peaks remained unidentified. Although rigorous filtering was applied, unidentified peaks should be interpreted cautiously until confirmed by retention index or standard validation. Moreover, because the cultivars were collected from their customary production areas, genotype and environmental factors are inherently confounded, indicating that the observed differences likely reflect genotype × environment interactions rather than pure cultivar effects. Furthermore, the small number of biological replicates (*n* = 3) limits the robustness of conclusions for breeding applications. The potential influence of cooking and matrix effects, particularly for lipid-derived compounds such as 1-octen-3-ol, should also be acknowledged.

Future studies should employ controlled environmental designs to disentangle genotype and environmental effects, increase biological replication to at least 5–7 field-derived samples and incorporate external standard validation and retention index data for key peaks. Integrating glycosylated precursor analysis and targeted sensory evaluation of key VOCs will be critical for linking chemical profiles to perceptual relevance. Systematic investigation of cooking and matrix effects will further refine the interpretation of lipid-derived VOCs and reduce the risk of artifacts. In addition, coupling GC × GC fingerprints with OAV analysis will help ensure that chemically minor but odor-active compounds are properly represented, ultimately supporting the development of more robust aroma-based breeding strategies for japonica rice.

## 4. Conclusions

This study qualitatively profiled 1924 volatile compounds in 11 non-aromatic japonica rice cultivars in Japan using GC × GC-TOFMS, revealing distinct VOC signatures that separated glutinous and normal lines. Normal-amylose rice contained more ethers, aldehydes and amines, whereas glutinous rice showed higher levels of hydrocarbons, ketones and acids, mirroring their contrasting sensory impressions. Given the qualitative identifications, the large proportion of unannotated peaks, the genotype-by-environment confounding and the limited replication (*n* = 3), these results serve as an initial framework for exploring aroma diversity in japonica rice.

## Figures and Tables

**Table 1:** Candidate rice cultivars used for examination.

No.	Cultivar	Amylose Content %	Area	Producing Year
1	Akitakomachi	20.0	Akita	2021
2	Masshigura	19.6	Aomori	2021
3	Nanatsuboshi	19.8	Hokkaido	2021
4	Hitomebore	20.1	Iwate	2021
5	Sasanishiki	19.7	Miyagi	2022
6	Hinohikari	19.4	Ooita	2022
7	Koshihikari	20	Uonuma, Niigata	2022
8	KitayukiMochi	0	Hokkaido	2021
9	Tatsukomochi	0	Akita	2021
10	Hiyokumochi	0	Saga	2021
11	KazenokoMochi	0	Hokkaido	2021

## Data Availability

The original contributions presented in this study are included in the article. Further inquiries can be directed to the corresponding author.

## Appendix A

**Table A1:** The top 50 component RSD values of Koshihikari (normal rice).

Name	Formula	Average/ Similarity	Average/F-Ratio	Koshihikari_01	Koshihikari_02	Koshihikari_03	RSD Value
3-exo-Chloro-6-endo-nitrobicyclo[2.2.1]heptan-2-one	C7H8ClNO3	832	392.86	4.66 × 10^7^	4.63 × 10^7^	4.89 × 10^7^	3.04
2,2-Bis(ethylsulfonyl)propane	C16H14Cl2O2	562	23.22	3.21 × 10^4^	6.56 × 10^4^	3.23 × 10^4^	44.49
p-Toluic acid, 3-pentadecyl ester	C16H26	776	6.04	3.22 × 10^2^	2.51 × 10^2^	2.83 × 10^2^	12.41
Cyclohexanone, 2-(2-nitro-2-propenyl)-	C11H20O	668	50.46	1.90 × 10^6^	2.12 × 10^6^	2.09 × 10^6^	5.83
Propanoic acid, 2-methyl-, 3-hydroxy-2,2,4-trimethylpentyl ester	C15H18N4O3S3	551	38.27	6.43 × 10^6^	6.51 × 10^6^	6.88 × 10^6^	3.66
Tetraborane(10)	C30H52O2	634.5	27.31	1.88 × 10^4^	3.23 × 10^4^	1.15 × 10^4^	50.56
Formic acid, hexyl ester	C5H6O	771	91.00	1.36 × 10^6^	1.57 × 10^6^	1.50 × 10^6^	7.38
1-[trans-4-(2-Iodoethyl)cyclohexyl]-trans-4-pentylcyclohexane	C21H42	791	21.19	1.52 × 10^5^	2.43 × 10^5^	1.38 × 10^5^	32.10
3,6,9,12-Tetraoxatetradecan-1-ol, 14-[4-(1,1,3,3-tetramethylbutyl)phenoxy]-	C15H17ClN2O3	550	8.66	7.88 × 10^2^	1.14 × 10^3^	1.82 × 10^3^	42.07
Furan, 2-pentyl-	C27H50O6	726.666667	36.74	6.76 × 10^4^	9.86 × 10^4^	1.34 × 10^5^	33.28
Isoamylalcohol	C20H30O2	696	34.76	6.38 × 10^3^	9.99 × 10^3^	6.44 × 10^3^	27.18
1H-Fluorene, dodecahydro-	C9H6O2	652	20.39	2.56 × 10^6^	1.88 × 10^6^	2.82 × 10^6^	20.07
Mannosamine	C16H32O3	722.75	49.03	2.82 × 10^7^	2.24 × 10^7^	3.41 × 10^7^	20.70
1,3,5-Trioxane, 2,4,6-trimethyl-	C23H46O3	664	20.46	2.27 × 10^5^	2.70 × 10^5^	2.37 × 10^5^	9.17
1,1-D2-2-(d3-methyl)-4-methyl-1-pentene	C4Cl3F7	619	33.71	8.56 × 10^2^	3.47 × 10^3^	5.97 × 10^2^	96.89
1-Hexadecanol	C19H30	576	10.36	4.32 × 10^5^	4.50 × 10^5^	6.10 × 10^5^	19.71
Benzenemethanol	C7H5NS	863	24.39	4.29 × 10^6^	7.36 × 10^6^	5.52 × 10^6^	26.96
Bicyclo[7.1.0]dec-2-yne	C4H8O2	868	52.29	3.48 × 10^6^	3.57 × 10^6^	3.94 × 10^6^	6.68
2,3-Octanedione	C16H24O2	612.5	14.41	1.12 × 10^6^	1.17 × 10^6^	1.18 × 10^6^	3.15
Nonane, 3-methyl-	C39H80O2	573	19.60	1.97 × 10^6^	2.13 × 10^6^	1.91 × 10^6^	5.56
(2R,3R)-2-(benzoxymethyl)-3-(chloromethyl)oxirane	C19H40	668	18.34	3.83 × 10^4^	3.48 × 10^4^	2.65 × 10^4^	18.26
Acetamide, N-(2,3-dichlorophenyl)-2-[(1-naphthalenylmethyl)amino]-	C20H32O3	613	18.00	8.03 × 10^3^	1.53 × 10^4^	8.81 × 10^3^	37.45
1-Hepten-3-ol	C9H9N	680	32.75	3.45 × 10^3^	8.53 × 10^3^	3.42 × 10^3^	57.39
3-Amino-2-oxazolidinone	C8H18O	576	29.99	2.78 × 10^5^	4.04 × 10^5^	2.80 × 10^5^	22.60
1-deoxy-D-ribitol	C31H64O	820.65	20.86	1.90 × 10^7^	2.87 × 10^7^	1.96 × 10^7^	24.15
2-Nonanone	C3H5FO	636	134.53	3.65 × 10^6^	3.63 × 10^6^	4.08 × 10^6^	6.61
Cyclohexane, 1,2,4,5-tetramethyl-, (1alpha±,2alpha±,4alpha±,5alpha±)-	C13H24	762	15.99	3.33 × 10^3^	1.01 × 10^4^	5.56 × 10^3^	54.45
2-Butyl-1-decene	C15H22O	731	24.60	8.48 × 10^4^	1.19 × 10^5^	7.21 × 10^4^	26.12
Lauric acid, 2-methylbutyl ester	C19H34O2	575	35.43	1.66 × 10^7^	1.69 × 10^7^	1.63 × 10^7^	1.77
4-Methyl-hexadecahydro-pyrene	C20H38	682	7.47	2.34 × 10^3^	4.22 × 10^3^	3.43 × 10^3^	28.26
1,2,4-Butanetriol	C20H32	724	109.54	3.52 × 10^7^	3.63 × 10^7^	3.42 × 10^7^	2.98
Cyclohexane, 1,2,3,5-tetraisopropyl-	C19H34	753.333333	28.80	1.10 × 10^7^	1.29 × 10^7^	9.98 × 10^6^	13.24
1,2-Dihydrothieno[3,4-d]pyridazine	C9H16	727	28.63	2.55 × 10^5^	2.72 × 10^5^	2.90 × 10^5^	6.38
Benzenepropanenitrile <4-ethyl-, alpha,alpha-, dimethyl->	C15H30O2	785	10.40	2.20 × 10^5^	1.75 × 10^5^	2.52 × 10^5^	17.82
Hexanal	ClHO	756.375	413.64	1.07 × 10^9^	1.07 × 10^9^	1.10 × 10^9^	1.50
15-Bromo-2-pentadecanone	C14H31N	691	80.56	9.49 × 10^4^	6.96 × 10^4^	6.29 × 10^4^	22.28
Isoamyl laurate	C26H44O4S	672	138.56	1.75 × 10^5^	4.09 × 10^5^	2.10 × 10^5^	47.60
4,6-Dimethylundecane	C9H16O	916	18.08	6.04 × 10^2^	1.10 × 10^3^	1.65 × 10^3^	46.76
Glycine, N-benzoyl-	C40H82O2	710	17.64	4.48 × 10^3^	1.64 × 10^4^	4.90 × 10^3^	78.67
1-Decene, 3,4-dimethyl-	C8H18O	799.857143	27.82	1.06 × 10^9^	1.26 × 10^9^	1.24 × 10^9^	9.26
1-Dodecanamine, N,N-dimethyl-	C12H20	594	107.94	2.25 × 10^7^	2.22 × 10^7^	2.25 × 10^7^	0.68
Acetamide, N-(6-acetylaminobenzothiazol-2-yl)-2-(adamantan-1-yl)-	C14H24	652	15.30	1.16 × 10^2^	2.58 × 10^2^	0	103.61
1-Dodecanol	C19H30	576	10.36	4.32 × 10^5^	4.50 × 10^5^	6.10 × 10^5^	19.71
3-Nonyn-1-ol	C15H12BrN3O2	639.285714	138.63	1.01 × 10^7^	1.27 × 10^7^	1.25 × 10^7^	12.22
Octanal	C17H36O	712	145.31	4.20 × 10^7^	4.42 × 10^7^	3.95 × 10^7^	5.58
Dodecanoic acid, 1,1-dimethylpropyl ester	C18H38O2	695	40.70	2.99 × 10^5^	3.43 × 10^5^	3.28 × 10^5^	6.83
Benzene, hexyl-	C4H8O	695.4	37.23	4.09 × 10^6^	4.19 × 10^6^	4.46 × 10^6^	4.55
Hexane, 1-nitro-	C27H30O9	570	27.63	1.84 × 10^4^	7.17 × 10^4^	4.97 × 10^4^	57.48
1-Pentanamine	C27H32ClNO2	611	188.34	2.40 × 10^6^	2.39 × 10^6^	2.65 × 10^6^	5.84
Butanal, 3-methyl-	C12H24	550	135.20	3.29 × 10^5^	3.88 × 10^5^	3.94 × 10^5^	9.64

**Table A2:** The top 50 components with the highest −log10 (0.05) of Koshihikari.

No.	Name	Formula	*m*/*z*	Area	RT1	RT2	−log10(*p* Value)	log2(FC)
1	3-exo-Chloro-6-endo-nitrobicyclo[2.2.1]heptan-2-one	C7H8ClNO3	189.02	4.04 × 10^6^	423.5339	0.8867	5.61	2.70
2	2,2-Bis(ethylsulfonyl)propane	C7H16O4S2	228.33	9.52 × 10^7^	544.5436	0.8334	5.17	2.51
3	p-Toluic acid, 3-pentadecyl ester	C23H38O2	346.50	8.58 × 10^5^	539.0431	1.7335	4.75	2.76
4	Cyclohexanone, 2-(2-nitro-2-propenyl)-	C9H13NO3	183.20	2.58 × 10^5^	467.5374	1.8568	4.62	2.45
5	Propanoic acid, 2-methyl-, 3-hydroxy-2,2,4-trimethylpentyl ester	C12H24O3	216.32	4.9 × 10^6^	1138.5911	1.3934	4.41	3.01
6	Tetraborane(10)	B4H10	53.32	1.71 × 10^5^	126.5101	3.3336	4.37	5.55
7	Formic acid, hexyl ester	C7H14O2	130.18	5.74 × 10^4^	555.5444	2.4202	4.35	2.13
8	1-[trans-4-(2-Iodoethyl)cyclohexyl]-trans-4-pentylcyclohexane	C19H35I	390.39	1.84 × 10^6^	709.5568	4.7470	4.23	8.69
9	3,6,9,12-Tetraoxatetradecan-1-ol, 14-[4-(1,1,3,3-tetramethylbutyl)phenoxy]-	C24H42O6	426.60	5.02 × 10^6^	1039.5832	1.0001	4.22	4.97
10	Furan, 2-pentyl-	C9H14O	138.21	3.24 × 10^3^	445.5356	1.3818	4.20	2.62
11	Isoamylalcohol	C5H12O	88.15	1.23 × 10^5^	456.5365	0.9934	4.19	3.44
12	1H-Fluorene, dodecahydro-	C13H22	178.31	1.59 × 10^6^	676.5541	2.8936	4.12	2.46
13	Mannosamine	C6H13NO5	179.17	1.2 × 10^5^	143.0114	3.6536	4.11	4.85
14	1,3,5-Trioxane, 2,4,6-trimethyl-	C6H12O3	132.16	4.26 × 10^3^	242.0194	2.3335	4.10	3.83
15	1,1-D2-2-(d3-methyl)-4-methyl-1-pentene	C7H9D5	103.14	1.02 × 10^9^	242.0194	1.4201	4.09	3.73
16	1-Hexadecanol	C16H34O	242.44	9.06 × 10^7^	1462.2003	2.2402	4.04	2.95
17	Benzenemethanol	C7H8O	108.14	5.65 × 10^6^	1133.0906	0.8734	4.04	4.81
18	Bicyclo[7.1.0]dec-2-yne	C10H14	134.22	1.03 × 10^3^	1303.6043	2.9602	4.00	2.48
19	2,3-Octanedione	C8H14O2	142.20	9.13 × 10^5^	517.0414	1.1934	3.99	2.47
20	Nonane, 3-methyl-	C10H22	142.28	3.68 × 10^4^	1078.0862	3.7336	3.92	3.96
21	(2R,3R)-2-(benzoxymethyl)-3-(chloromethyl)oxirane	C11H13ClO2	212.67	3.54 × 10^5^	940.5752	5.1737	3.88	2.95
22	Acetamide, N-(2,3-dichlorophenyl)-2-[(1-naphthalenylmethyl)amino]-	C19H16Cl2N2O	359.25	7.76 × 10^7^	1276.1021	1.3601	3.84	4.83
23	1-Hepten-3-ol	C7H14O	114.19	2.61 × 10^4^	643.5515	2.5002	3.81	4.85
24	3-Amino-2-oxazolidinone	C3H6N2O2	102.09	2.71 × 10^4^	269.5216	3.8003	3.80	3.95
25	1-deoxy-D-ribitol	C5H12O4	136.15	1.22 × 10^6^	236.5189	0.8401	3.78	2.34
26	2-Nonanone	C9H18O	142.24	7.18 × 10^7^	583.0466	1.3934	3.76	2.87
27	Cyclohexane, 1,2,4,5-tetramethyl-, (1alpha±,2alpha±,4alpha±,5alpha±)-	C10H20	140.27	2.62 × 10^5^	280.5224	1.7735	3.74	3.22
28	2-Butyl-1-decene	C14H28	196.37	2.31 × 10^6^	588.5471	3.6603	3.74	2.24
29	Lauric acid, 2-methylbutyl ester	C17H34O2	270.45	1.18 × 10^7^	1390.2362	2.8736	3.74	6.53
30	4-Methyl-hexadecahydro-pyrene	C17H28	232.40	1.73 × 10^6^	1298.1038	3.0069	3.72	2.10
31	1,2,4-Butanetriol	C4H10O3	106.12	3.69 × 10^3^	299.7740	3.3536	3.69	4.29
32	Cyclohexane, 1,2,3,5-tetraisopropyl-	C18H36	252.50	6.42 × 10^3^	778.3123	5.1004	3.66	8.80
33	1,2-Dihydrothieno[3,4-d]pyridazine	C6H6N2S	138.19	7.39 × 10^7^	429.0343	2.0402	3.66	2.34
34	Benzenepropanenitrile <4-ethyl-, alpha,alpha-, dimethyl->	C13H17N	187.28	2.69 × 10^6^	2334.0200	1.1334	3.64	3.52
35	Hexanal	C6H12O	100.16	3.47 × 10^4^	368.5295	1.2157	3.63	4.57
36	15-Bromo-2-pentadecanone	C15H29BrO	304.14	1.99 × 10^5^	1556.6245	2.3269	3.63	7.97
37	Isoamyl laurate	C17H34O2	270.50	1.76 × 10^8^	1393.4448	2.8513	3.60	6.34
38	4,6-Dimethylundecane	C13H28	184.36	2.38 × 10^5^	330.0264	3.3269	3.57	3.56
39	Glycine, N-benzoyl-	C9H9NO3	179.17	2.83 × 10^6^	1727.1382	0.7934	3.55	4.17
40	1-Decene, 3,4-dimethyl-	C12H24	168.32	1.64 × 10^4^	445.5356	2.9802	3.54	2.93
41	1-Dodecanamine, N,N-dimethyl-	C14H31N	213.40	3.35 × 10^5^	836.0669	4.5270	3.54	2.76
42	Acetamide, N-(6-acetylaminobenzothiazol-2-yl)-2-(adamantan-1-yl)-	C21H25N3O2S	383.50	1.52 × 10^7^	610.5488	0.8867	3.50	2.26
43	1-Dodecanol	C12H26O	186.33	1.03 × 10^8^	1190.8453	1.8379	3.48	4.29
44	3-Nonyn-1-ol	C9H16O	140.22	1.31 × 10^5^	467.5374	1.5935	3.46	3.40
45	Octanal	C8H16O	128.21	8.47 × 10^8^	485.4138	1.4384	3.43	2.40
46	Dodecanoic acid, 1,1-dimethylpropyl ester	C17H34O2	270.50	5.73 × 10^3^	1386.1109	3.7003	3.42	7.66
47	Benzene, hexyl-	C12H18	162.27	2.52 × 10^3^	731.5585	1.8535	3.42	3.89
48	Hexane, 1-nitro-	C6H13NO2	131.17	5.3 × 10^6^	693.0554	1.1468	3.41	3.86
49	1-Pentanamine	C5H13N	87.16	1.02 × 10^7^	511.5409	0.9334	3.39	2.92
50	Butanal, 3-methyl-	C5H10O	86.13	2.19 × 10^4^	253.0202	1.2368	3.37	2.52

**Table A3:** The top 50 components with the highest −log10 (0.05) common to the four types of glutinous rice.

No.	Name	Formula	*m*/*z*	Area	RT1	RT2	−log10(*p* Value)	log2(FC)
1	2,3-dihydro-2(S),5-dimethyl-1,5-benzoxazepin-4(5H)-thione	C11H13NOS	207.07	3.89 × 10^4^	1529.1223	4.6537	5.42	−4.61
2	N,N-Dimethyl-1-decanamine	C12H27N	185.35	2.58 × 10^3^	819.5656	3.8970	4.81	−4.40
3	Undecane, 4-ethyl-	C13H28	184.36	5.73 × 10^2^	654.5524	4.4004	4.40	−6.62
4	1-Heptadecanol	C17H36O	256.50	2.54 × 10^2^	621.5497	3.8536	4.32	−6.51
5	Methyl 2-(1,3-dioxo-1,3-dihydro-2H-isoindol-2-yl)propanoate	C12H11NO4	233.22	4.29 × 10^5^	1688.6351	3.1936	4.31	−4.86
6	Methanediamine, N,N,N’,N’-tetrabutyl-	C17H38N2	270.50	9.70 × 10^5^	308.0246	1.5646	4.24	−4.91
7	Benzoic acid, tetradecyl ester	C21H34O2	318.50	8.28 × 10^4^	2040.6632	2.1113	4.16	−4.13
8	(1R,3aS,8aS)-7-Isopropyl-1,4-dimethyl-1,2,3,3a,6,8a-hexahydroazulene	C15H24	204.35	9.26 × 10^6^	1045.0836	2.8136	4.13	−5.91
9	(3E)-3-Icosene	C20H40	280.50	1.82 × 10^6^	693.0554	4.6070	4.11	−3.47
10	1,7,7-trimethylbicyclo[2.2.1]heptan-2-one	C10H16O	152.23	6.82 × 10^1^	665.5532	1.2201	4.00	−6.22
11	2-Methyl-E,E-3,13-octadecadien-1-ol	C19H36O	280.50	1.58 × 10^4^	2057.1646	2.0802	4.00	−4.31
12	Benzoic acid, tridecyl ester	C20H32O2	304.50	1.17 × 10^4^	2046.1637	2.1135	3.98	−4.50
13	Propane, 2,2’-[ethylidenebis(oxy)]bis-	C8H18O2	146.23	1.20 × 10^3^	2541.2033	2.8869	3.97	−3.88
14	Dichloroacetic acid, 2-tridecyl ester	C15H28Cl2O2	311.30	5.86 × 10^5^	1358.6087	1.3134	3.83	−4.31
15	3-(3-(Naphthalen-2-yl)propa-1,2-dienyl)thiophene	C17H12S	248.07	2.83 × 10^4^	2010.4108	2.2668	3.78	−4.21
16	2,5-Furandione, 3-dodecyl-	C16H26O3	266.38	7.88 × 10^1^	1331.1065	5.1737	3.78	−8.29
17	Dichloroacetic acid, 3-tetradecyl ester	C16H30Cl2O2	325.30	1.09 × 10^7^	682.0546	5.1971	3.66	−4.11
18	Heneicosane, 5-methyl-	C22H46	310.60	3.16 × 10^2^	1289.8532	5.2138	3.62	−3.67
19	Sulfurous acid, butyl cyclohexylmethyl ester	C11H22O3S	234.36	7.58 × 10^1^	885.5708	4.3203	3.36	−4.98
20	Benzo[k]fluoranthene-2,3-dione	C20H10O2	282.30	6.29 × 10^4^	2700.7160	4.1403	3.31	−5.72
21	Cyclohexane, undecyl-	C17H34	238.50	5.23 × 10^2^	1287.1030	4.2470	3.30	−3.06
22	(9E,11E)-Octadecadienoic acid	C18H32O2	280.40	2.18 × 10^5^	2640.2112	1.5468	3.28	−3.64
23	Tetracosane <n->	C24H50	338.70	9.57 × 10^3^	1168.2935	0.5854	3.20	−3.89
24	(3R,13R)-3,13-Dimethylheptadecane	C19H40	268.50	1.92 × 10^5^	819.5656	0.3867	3.16	−11.61
25	1,3,3-Trimethyl-2-(2-methyl-cyclopropyl)-cyclohexene	C13H22	178.31	7.54 × 10^5^	610.5488	2.6869	3.14	−2.58
26	1-Methyl-2-(4-methylpentyl)cyclopentane	C12H24	168.32	1.19 × 10^5^	418.0334	2.5269	3.08	−2.79
27	cis-4a-Methyl-decahydronaphthalene	C11H20	152.28	6.45 × 10^2^	786.5629	2.8036	3.08	−5.63
28	Ginsenol	C15H26O	222.37	2.71 × 10^5^	759.0607	3.6536	3.04	−6.47
29	Benzene, 1,3-bis(2-ethoxypropyl)-	C16H26O2	250.38	4.60 × 10^4^	2805.2244	3.9737	2.98	−2.29
30	Cyclododecylamine	C12H25N	183.33	3.70 × 10^6^	984.5788	3.0936	2.97	−4.02
31	Tetracosane	C24H50	338.70	1.38 × 10^3^	1136.7576	1.7312	2.96	−4.04
32	3-Ethyl-3-methylnonadecane	C22H46	310.60	1.2 × 10^8^	891.0713	5.3604	2.92	−3.43
33	2-Propanol, 1-propoxy-	C6H14O2	118.17	5.52 × 10^4^	1694.1355	1.6001	2.90	−3.38
34	1,3,12-Nonadecatriene	C19H34	262.50	1.41 × 10^6^	1182.5946	4.0603	2.89	−4.68
35	Methyl 2,2-dimethyldecanoate	C13H26O2	214.34	2.51 × 10^1^	1424.6140	0.8867	2.84	−8.44
36	Isochiapin B	C19H22O6	346.38	3.90 × 10^3^	561.0449	3.3003	2.83	−3.57
37	Cyclododecanepentanoic acid, 1-nitro-beta,2-dioxo-, phenylmethyl ester	C24H33NO6	431.23	1.20 × 10^6^	1369.6096	1.0734	2.81	−3.43
38	6-Dodecene, (Z)-	C12H24	168.32	6.87 × 10^3^	434.5348	2.4535	2.76	−6.26
39	D:A-Friedooleanan-7-ol, (7alpha±)-	C30H52O	428.70	2.66 × 10^3^	1474.1179	4.3804	2.76	−3.58
40	Propane, 2-bromo-1-chloro-	C3H6BrCl	157.44	6.35 × 10^2^	643.5515	4.9337	2.73	−4.24
41	Tetracontane	C40H82	563.10	1.65 × 10^2^	1424.6140	0.9267	2.73	−5.66
42	2,6-Di-tert-butyl-4-nitrophenol	C14H21NO3	251.32	5.08 × 10^3^	2046.1637	1.2734	2.70	−3.70
43	2,10-Phenanthrenediol, 4b,5,6,7,8,8a,9,10-octahydro-4b,8,8-trimethyl-1-(1-methylethyl)-, [4bS-(4balpha±,8abeta,10beta)]-	C20H30O2	302.46	7.10 × 10^2^	2332.1866	2.0335	2.70	−4.07
44	1-Nonanol	C9H20O	144.25	9.94 × 10^6^	913.0730	1.1268	2.60	−3.77
45	8-endo-Methylbicyclo[4.2.0]oct-2-ene	C9H14	122.21	5.72 × 10^3^	995.5796	4.8337	2.59	−3.04
46	5-Amino-2-methyl-2-phenyl-2,3-dihydro[1,2,4]triazolo[1,5-a][1,3,5]triazine	C11H12N6	228.11	2.23 × 10^3^	2128.6703	1.7801	2.58	−3.29
47	(3E)-1,4-diphenyl-3-buten-2-ol	C16H16O	224.30	5.21 × 10^5^	2095.6676	1.8135	2.56	−2.89
48	3,4-Dimethylbenzophenone	C15H14O	210.27	8.06 × 10^3^	2079.1663	1.2468	2.50	−3.27
49	Butanoic acid, 4-methoxy-	C5H10O3	118.13	8.16 × 10^6^	2612.7090	0.3867	2.50	−5.84
50	16-Hentriacontanone	C31H62O	450.80	8.30 × 10^6^	2497.1998	4.5248	2.49	−5.57
